# MiR‐629 regulates hypoxic pulmonary vascular remodelling by targeting FOXO3 and PERP

**DOI:** 10.1111/jcmm.14385

**Published:** 2019-06-26

**Authors:** Mei Zhao, Ni Chen, Xuelian Li, Ling Lin

**Affiliations:** ^1^ Department of Pharmacy Sanya Central Hospital The Third People’s Hospital of Hainan Province Sanya China; ^2^ Department of Pharmacy The Second Affiliated Hospital of Hainan Medical University Haikou China; ^3^ Department of Pharmacology, College of Pharmacy Harbin Medical University Harbin China; ^4^ Department of Cardiovascular Medicine Sanya Central Hospital The Third People’s Hospital of Hainan Province Sanya China

**Keywords:** FOXO3, hypoxia, miR‐629, PAH, PERP, PHASMCs

## Abstract

Pulmonary arterial hypertension (PAH) is featured by the increase in pulmonary vascular resistance and pulmonary arterial pressure. Despite that abnormal proliferation and phenotypic changes in human pulmonary artery smooth muscle cells (HPASMCs) contributing to the pathophysiology of PAH, the underlying molecular mechanisms remain unclear. In the present study, we detected the expression of miR‐629 in hypoxia‐treated HPASMCs and explored the mechanistic role of miR‐629 in regulating HPASMC proliferation, migration and apoptosis. Hypoxia time‐dependently induced up‐regulation of miR‐629 and promoted cell viability and proliferation in HPASMCs. Treatment with miR‐629 mimics promoted HPASMCs proliferation and migration, but inhibited cell apoptosis; while knockdown of miR‐629 suppressed the cell proliferation and migration but promoted cell apoptosis in HPASMCs. The bioinformatics prediction revealed FOXO3 and PERP as downstream targets of miR‐629, and miR‐629 negatively regulated the expression of FOXO3 and PERP via targeting the 3’ untranslated regions. Enforced expression of FOXO3 or PERP attenuated the miR‐629 overexpression or hypoxia‐induced enhanced effects on HPASMC proliferation and proliferation, and the suppressive effects on HPASMC apoptosis. Furthermore, the expression of miR‐629 was up‐regulated, and the expression of FOXO3 and PERP mRNA was down‐regulated in the plasma from PAH patients when compared to healthy controls. In conclusion, the present study provided evidence regarding the novel role of miR‐629 in regulating cell proliferation, migration and apoptosis of HPASMCs during hypoxia.

## 1 INTRODUCTION

Pulmonary arterial hypertension (PAH) is featured by the increase in pulmonary vascular resistance and pulmonary arterial pressure that often lead to right heart dysfunction and is regarded as a devastating and life‐threatening disease.^1^, ^2^ Although great efforts have been made to determine the pathophysiology of PAH, the specific molecular mechanisms of PAH remain elusive. Multiple lines of evidence suggested that the vascular remodelling by abnormal proliferation and phenotypic changes in human pulmonary artery smooth muscle cells (HPASMCs) contribute to the genesis and development of PAH.^3^, ^4^, ^5^ Targeting vascular remodelling has been proposed as a strategy for the development novel therapies for PAH.^6^ Therefore, it is necessary for us to understand the molecular mechanisms underlying the HPASMCs‐mediated vascular remodelling.

MicroRNAs (miRNAs) are a class of small non‐coding RNAs with 21‐23 nucleotides in length, and miRNAs exerted the biological functions via targeting the 3′ untranslated region (3′UTR) of the genes to transcriptionally repress the gene expression.^7^, ^8^ miRNAs are found to involve in many biological processes including cell proliferation, apoptosis, invasion, migration and metabolism.^9^ To date, a large body of evidence has pointed to the functional role of miRNAs in the pathophysiology of PAH. Caruso et al showed that miR‐124 was down‐regulated in the pulmonary vascular and circulating progenitor endothelial cells from PAH patients, and dysregulation of miR‐124 contributed to the endothelial cell glycolysis.^10^ miR‐4224 and miR‐503 were found to mediate the link between endothelial apelin and fibroblast growth factor 2, which was disrupted in the PAH.^11^ In addition, down‐regulation of miR‐126 contributed to the right ventricle failure in PAH and restoration miR‐129 expression in the right ventricle improved micro‐vessel density and right ventricle function in experimental PAH.^12^ miR‐629 was a newly identified miRNA and plays an oncogenic role in several types of cancers including cervical cancer, colorectal cancer, breast cancer and ovarian cancer.^13^, ^14^, ^15^, ^16^ Despite the role of miR‐629 in cancer, the role of miR‐629 in PAH has not been investigated so far.

In this study, we identified the up‐regulation of miR‐629 in the HPASMCs under hypoxia conditions, and further in vitro functional studies showed that miR‐629 exerted enhanced effects on the proliferation of HPASMCs, and mechanistic investigations demonstrated that miR‐629 exerted its effects on cellular functions via regulating forkhead box O3 (FOXO3) and p53 apoptosis effector related to PMP‐22 (PERP) in HPASMCs. The evidence in the present study provided a novel role of miR‐629 in the hyper‐proliferated HPASMCs, which may be associated with the development of PAH.

## 2 MATERIALS AND METHODS

### 2.1 Cell culture

The HPASMCs were obtained from Sigma‐Aldrich (St. Louis, USA). The cells were cultured in the smooth muscle cell growth medium (Sigma‐Aldrich) supplemented with 10% foetal bovine serum (FBS; Thermo Fisher Scientific, Waltham, USA). The cells were maintained in a humidified incubator with 5% CO_2_ at 37°C.

### 2.2 Hypoxia treatment, oligo nucleotides and cell transfections

For the hypoxia treatment, HPASMCs were incubated in a gastight molecular incubator chamber (Thermo Fisher Scientific) supplied with 3% O_2_, 5% CO_2_ and 92% N_2_ for 12, 24 or 48 hours, respectively, and treated cells were collected for further in vitro assays. The miRNAs including miR‐629 mimics, miR‐629 inhibitors as well as the negative controls (NCs; mimics NC and inhibitors NC) were purchased from Ribobio (Guangzhou, China). The FOXO3 or PERP‐overexpressing vectors were constructed by cloned the pcDNA of FOXO3 or PERP into the pcDNA3.1 vector (pcDNA3.1‐FOXO3 or pcDNA3.1‐PERP) and pcDNA3.1 was served as NC. For the cell transfections, HPASMCs were transfected with miRNAs or pcDNA3.1 constructs by using Lipofectamine 2000 reagent (Invitrogen, Carlsbad, USA) according to the manufacturer's protocol. At 24 hours post‐transfection, cells were collected for further in vitro assays.

### 2.3 Collection of plasma samples

A total of 14 PAH patients and 14 healthy volunteers were included in the study. The plasma samples were collected from these PAH patients and healthy controls at the Second Affiliated Hospital of Hainan Medical University between January 2016 and December 2017. The collected plasma samples were immediately stored in −80°C until use. The PAH patients (eight male and six female patients) had a mean age of 39.4 ± 6.7 and a mean pulmonary arterial pressure of 76.3 ± 12.3 mm Hg. The recruited patients had not received any local or systemic treatments. Written informed consent was obtained from each included patient and the study was approved by the Ethics Committee of the Second Affiliated Hospital of Hainan Medical University.

### 2.4 Quantitative real‐time PCR

Total RNA from cells was extracted by using the TRIzol reagent (Invitrogen) according to the manufacturer's protocol. For the quantification of miR‐629, cDNA was synthesized by the One Step Prime script miRNA cDNA synthesis kit (Qiagen, Valencia), and real‐time PCR was performed using the miRNA‐specific TaqMan MiRNA Assay Kit (Applied Biosystems, Foster City, USA) on an ABI7900 system (Applied Biosystems). For the mRNA detection, mRNA was reversely transcribed into cDNA using PrimeScript RT reagent kit (Takara, Dalian, China), and real‐time PCR was performed using SYBR Green Premix Ex Taq II kit (Takara) on an ABI7900 system (Applied Biosystems). U6 and GAPDH were used as internal controls for miRNA and mRNA expression, respectively, and the relative expression of respective genes was calculated by 2^−ΔΔCt^ method.

### 2.5 Western blot

Proteins from cells were extracted by the RIPA buffer (Bio‐Rad, Hercules) supplemented with protease inhibitors (Sigma‐Aldrich), and the concentrations of the extracted proteins were measured by BCA kit (Bio‐Rad) according to the manufacturer's protocol. The equal amounts of proteins were resolved on a 10% SDS‐PAGE, and after gel electrophoresis, the separated proteins were then transferred to the PVDF membranes. After being washed with PBST for three times, the membranes were incubated with 5% skimmed milk at room temperature for 1 hour, and the membranes were then subjected to incubation with different antibodies against proliferating cell nuclear antigen (PCNA), cleaved caspase‐3, FOXO3, PERP and β‐actin (Abcam, Cambridge) at 4°C overnight. Then, the membranes were washed with PBST for three times before incubating with the horseradish peroxidase‐conjugated secondary antibodies (Abcam). After being washed with PBST for three times, the blots were detected by an enhanced chemiluminescence detection system (Bio‐Rad) according to the manufacturer's protocol.

### 2.6 Cell counting kit‐8 assay

Cell viability of HPASMCs was detected by CCK‐8 assay (Beyotime, Beijing, China). Briefly, the treated HPASMCs were incubated with 10 μl CCK‐8 reagent for 2 hours at room temperature. The absorbance at a wavelength of 450 nm was determined by a microplate reader to assess the cell viability.

### 2.7 Bromodeoxyuridine assay

Cell proliferation of HPASMCs was determined using the BrdU assay kit (Millipore, Billerica, USA) according to the manufacturer's protocol. Briefly, the treated HPASMCs were incubated with 10 μl BrdU reagent at room temperature for 5 hours followed by fixing with FixDeant for 30 minutes. Cells were then incubated with anti‐BrdU antibody (Abcam) at 4°C overnight, followed by treatment with Cy5‐conjugated secondary antibody for 2 hours. Cell nuclei were stained with 4',6‐diamidino‐2‐phenylindole (Sigma‐Aldrich) for 15 minutes at room temperature. The BrdU activity was determined using a fluorescence confocal microscopy.

### 2.8 Cell migration assay

Cell migratory potential of HPASMCs was measured by transwell migration assay. Briefly, treated HPASMCs were seeded onto the upper chamber with inserts (8 μm pore size; Costar, Cambridge, USA) containing smooth muscle cell growth medium without FBS; while the lower chamber was filled with smooth muscle cell growth medium supplemented with 10% FBS as chemoattractant. After incubation for 24 hours, the cells on the upper surface membrane were cleaned by a cotton swab and the migrated cells were then fixed with 70% ethanol and stained with 0.5% crystal violet for 30 minutes at room temperature. The number of migrated cells was counted (five random fields per chamber) under a light microscope.

### 2.9 Caspase‐3 activity

For the measurement of the caspase‐3 activity, the treated HPASMCs were subjected to caspase‐3 activity determination using the Caspase‐3 Assay Kit (Abcam) according to manufacturer's protocol.

### 2.10 Luciferase reporter assay

The pGL3 vector (Promega, Madison, SUA) was used to construct the reporter vectors, and the fragment of wild‐type 3′UTR of FOXO3 or PERP containing the putative miR‐629 binding site was amplified from human genomic DNA and cloned into the downstream of the pGL3 vector, For the mutant, point mutations were introduced into the seed region of miR‐629 bindings sties. For the luciferase reporter assay, cells were co‐transfected with wild‐type or mutant reporter vectors, respective miRNAs (mimics NC or miR‐629 mimics) and pRL‐TK renilla luciferase reporter vector (Promega) by Lipofectamine 200 reagent (Invitrogen) according to the manufacturer's protocol. Relative luciferase activities were determined by Dual‐Luciferase Reporter Assay system (Promega) at 48 hours after co‐transfections.

### 2.11 Statistical analysis

All the data were expressed as mean ± standard deviation. GraphPad Prism 6.0 (GraphPad Software, La Jolla) was used to perform the statistical analysis and graph plotting. Student's *t* test and one‐way ANOVA were used for difference comparison between two groups or among more than two groups. Statistically significant differences were defined as *P* < 0.05.

## 3 RESULTS

### 3.1 Hypoxia‐induced miR‐629 up‐regulation, promoted cell viability and proliferation in HPASMCs

**Figure 1:**
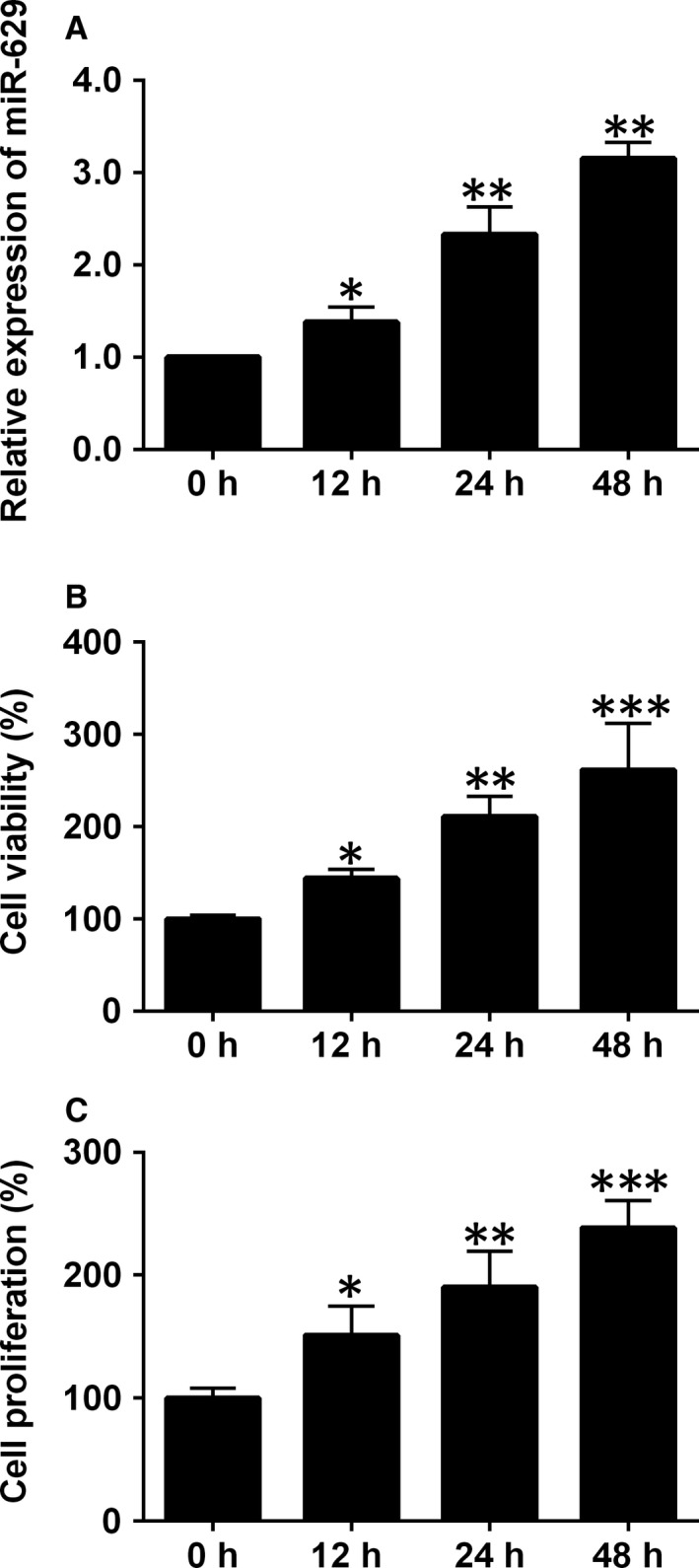
The effects of hypoxia on miR‐629 expression, cell viability and cell proliferation in HPASMCs. A, The expression of miR‐629 in HPASMCs after being treated with hypoxia for 12, 24 and 48 h was determined by qRT‐PCR. B, Cell viability of HPASMCs after being treated with hypoxia for 12, 24 and 48 h was determined by CCK‐8 assay. C, Cell proliferation of HPASMCs after being treated with hypoxia for 12, 24 and 48 h was determined by BrdU assay. Data are expressed as mean ± standard deviation; n = 3. *P < 0.05, **P < 0.01 and ***P < 0.001 versus control groups

To address the effects of hypoxia on the miR‐629 expression, cell viability and proliferation of HPASMCs, the HPASMCs were exposed to hypoxia for 12, 24 and 48 hours respectively. After hypoxia exposure, HPASMCs were subjected to qRT‐PCR assay, CCK‐8 assay and BrdU assay. As shown in Figure 1A, hypoxia treatment significantly up‐regulated the expression of miR‐629 compared to control group (Figure 1A), and this effect was time‐dependent. Consistently, hypoxia also time‐dependently increased the cell viability and proliferation of HPASMCs (Figure 1B,C). As 48 hours hypoxia exposure had the most profound effects, this time duration was selected for the following experiments.

### 3.2 Overexpression of miR‐629 promoted cell proliferation, cell migration and inhibited cell apoptosis of HPASMCs

**Figure 2:**
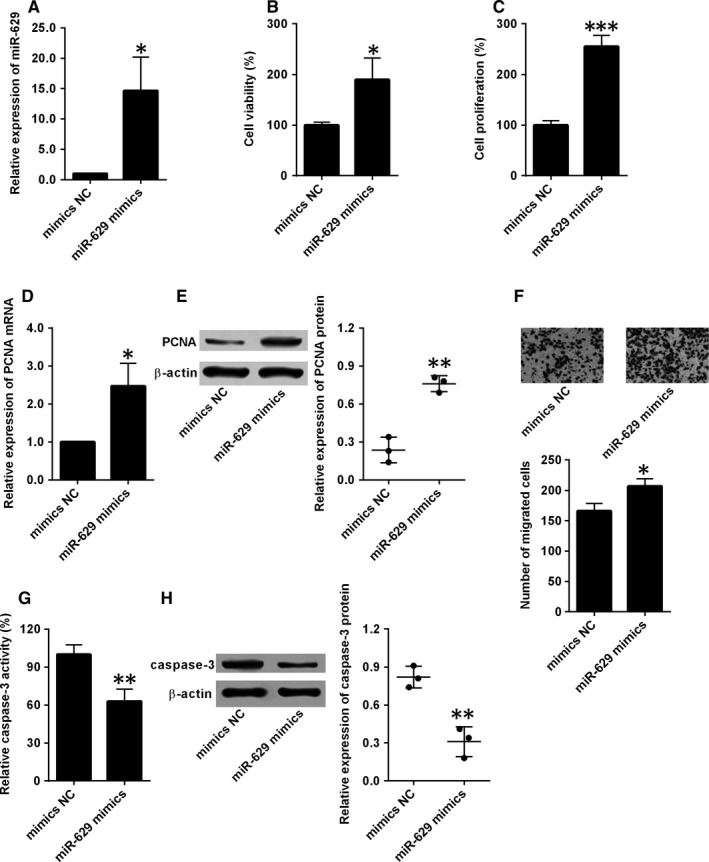
Overexpression of miR‐629 promoted cell proliferation, cell migration and inhibited cell apoptosis of HPASMCs. A, The expression of miR‐629 in HPASMCs after being transfected with mimics NC or miR‐629 mimics was determined by qRT‐PCR. B, Cell viability and (C) cell proliferation of HPASMCs after being transfected with mimics NC or miR‐629 mimics was detected by CCK‐8 assay and BrdU assay respectively. (D and E) The expression of PCNA mRNA and protein in HPASMCs after being transfected with mimics NC or miR‐629 mimics were determined by qRT‐PCR and western blot respectively. (F) Cell migration of HPASMCs after being transfected with mimics NC or miR‐629 mimics was determined by transwell migration assay. (G and H) The caspase‐3 activity and the expression of caspase‐3 protein in HPASMCs after being transfected with mimics NC or miR‐629 mimics were determined by Caspase‐3 activity assay and western blot respectively. Data are expressed as mean ± standard deviation; n = 3. ^*^ *P* < 0.05, ^**^ *P* < 0.01 and ^***^ *P* < 0.001 versus control groups

To further determine the functional role of miR‐629 in HPASMCs, overexpression of miR‐629 in HPASMCs was done by transfecting cells with miR‐629 mimics, which was confirmed by qRT‐PCR (Figure 2A). The effects of miR‐629 overexpression on the cell proliferation, migration and apoptosis were evaluated by the in vitro assays. The CCK‐8 and BrdU assays showed that HPASMCs transfected with miR‐629 mimics showed an enhanced cell viability and proliferation when compared to mimics NC group (Figure 2B,C). The qRT‐PCR and western blot consistently revealed that overexpression of miR‐629 induced the up‐regulation of PCNA mRNA and protein in HPASMCs (Figure 2D,E). For the transwell migration assay, the number of migrated cells was also significantly increased in miR‐629 mimics‐transfected HPASMCs compared to mimics NC group (Figure 2F). The effects of miR‐629 on cell apoptosis were evaluated by caspase‐3 activity and caspase‐3 protein expression, and overexpression of miR‐629 suppressed the caspase‐3 activity and caused a decreased in the caspase‐3 protein level (Figure 2G,H).

### 3.3 Knockdown of miR‐629 suppressed cell proliferation, cell migration and induced cell apoptosis of HPASMCs during hypoxia

**Figure 3:**
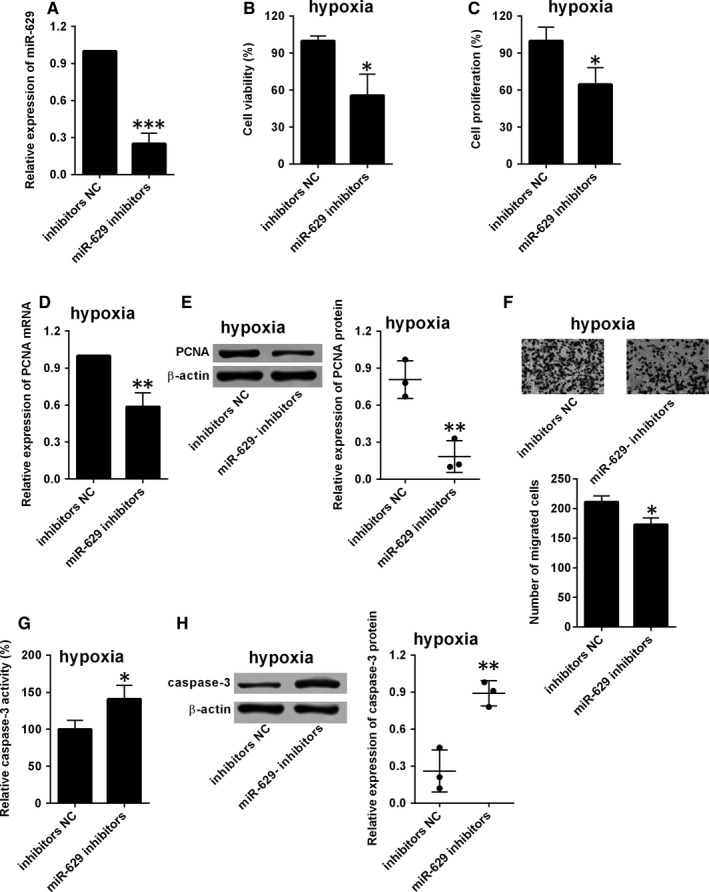
Knockdown of miR‐629 suppressed cell proliferation, cell migration and induced cell apoptosis of HPASMCs during hypoxia. A, The expression of miR‐629 in HPASMCs after being transfected with inhibitors NC or miR‐629 inhibitors was determined by qRT‐PCR. B, Cell viability and (C) cell proliferation of hypoxia‐treated HPASMCs after being transfected with inhibitors NC or miR‐629 inhibitors was detected by CCK‐8 assay and BrdU assay respectively. (D and E) The expression of PCNA mRNA and protein in hypoxia‐treated HPASMCs after being transfected with inhibitors NC or miR‐629 inhibitors were determined by qRT‐PCR and western blot respectively. F, Cell migration of hypoxia‐treated HPASMCs after being transfected with inhibitors NC or miR‐629 inhibitors was determined by transwell migration assay. (G and H) The caspase‐3 activity and the expression of caspase‐3 protein in hypoxia‐treated HPASMCs after being transfected with inhibitors NC or miR‐629 inhibitors were determined by Caspase‐3 activity assay and western blot respectively. Data are expressed as mean ± standard deviation; n = 3. ^*^ *P* < 0.05, ^**^ *P* < 0.01 and ^***^ *P* < 0.001 versus control groups

The effects of miR‐629 knockdown in hypoxia‐treated HPAMSCs were also determined by performing in vitro functional assays. As shown in Figure 3A, transfection with miR‐629 inhibitors significantly suppressed the expression of miR‐629 in HPASMCs compared to mimics NC transfection (Figure 3A). Furthermore, HPASMCs were first exposed to hypoxia for 48 h, and then the effects of miR‐629 on cell proliferation, migration and apoptosis were assessed. As revealed by CCK‐8 and BrdU assays, knockdown of miR‐629 significantly suppressed cell viability and proliferation of hypoxia‐treated HPASMCs (Figure 3B,C). In addition, mRNA and protein expression levels of PCNA in hypoxia‐treated HPASMCs were significantly suppressed by miR‐629 inhibitors transfection compared to inhibitors NC group (Figure 3D,E). The transwell migration assay showed that knockdown of miR‐629 also reduced the migratory potential of hypoxia‐treated HPASMCs (Figure 3F). In terms of cell apoptosis, knockdown of miR‐629 increased the caspase‐3 activity and protein level of caspase‐3 in hypoxia‐treated HPASMCs (Figure 3G,H).

### 3.4 MiR‐629 directly targets FOXO3 and PERP

**Figure 4:**
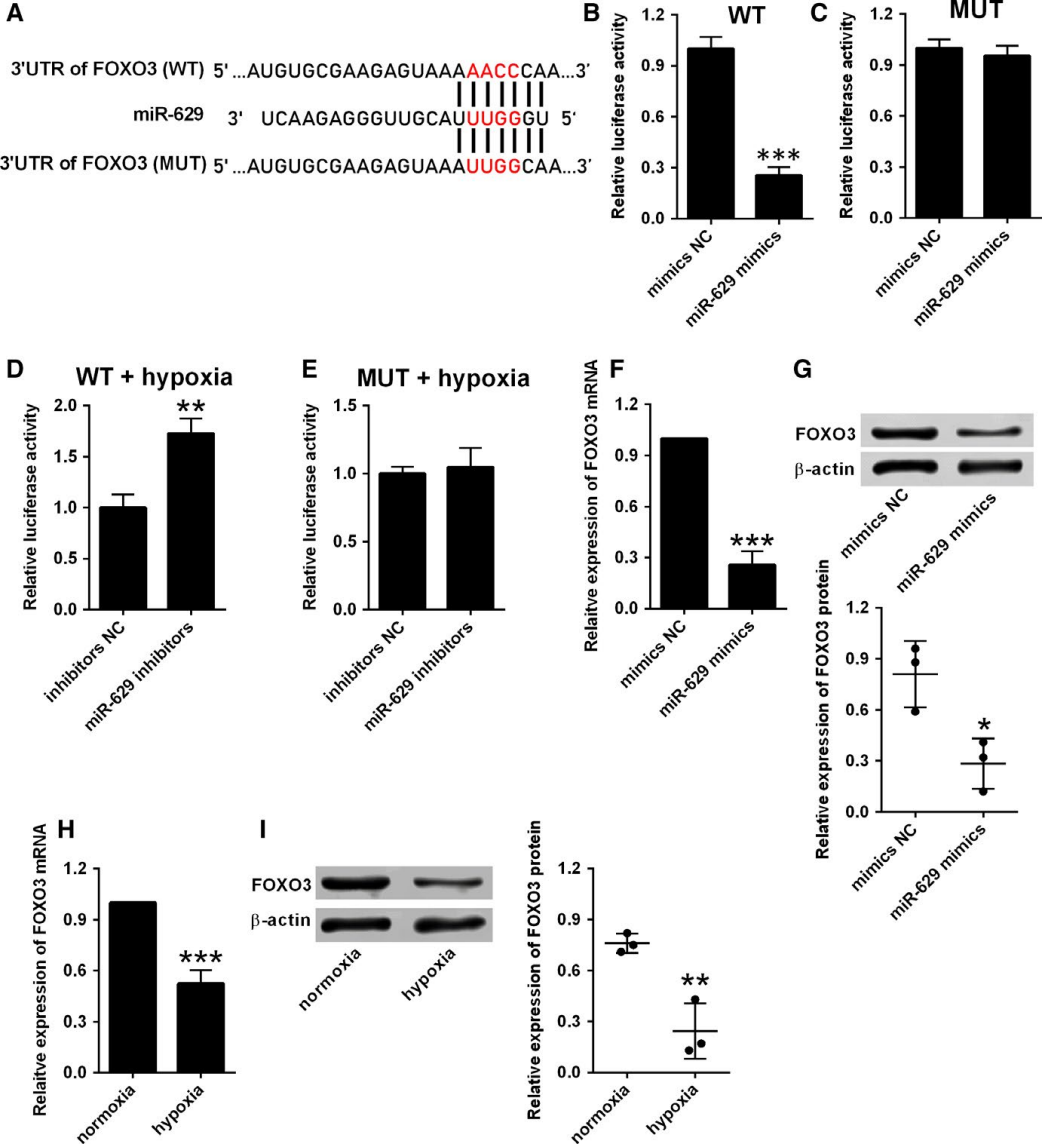
miR‐629 directly targets FOXO3. A, The predicted binding sites between 3′UTR of FOXO3 and miR‐629 as determined by the targetscan software. B, The relative luciferase activity of the reporter vector containing wide‐type (WT) 3′UTR of FOXO3 was determined at 48 h after transfection with mimics NC or miR‐629 mimics. C, The relative luciferase activity of the reporter vector containing mutant (MUT) 3′UTR of FOXO3 was determined at 48 h after transfection with mimics NC or miR‐629 mimics. (D and E) The relative luciferase activity of the reporter vector containing mutant 3′UTR of FOXO3 was determined at 48 h after transfection with inhibitors NC or miR‐629 inhibitors in hypoxia‐treated HPASMCs. (F and G) The expression of FOXO3 mRNA and protein in HPASMCs after being transfected with mimics NC or miR‐629 mimics was determined by qRT‐PCR and western blot respectively. (H and I) The expression of FOXO3 mRNA and protein in HPASMCs after exposing to hypoxia or normoxia was determined by qRT‐PCR and western blot respectively. Data are expressed as mean ± standard deviation; n = 3. *P < 0.05, **P < 0.01 and ***P < 0.001 versus control groups

To address the mechanistic role of miR‐629 in HPASMCs, we performed the bioinformatics prediction analysis using the targetscan software. Among these predicted targets, FOXO3 and PERP were further selected for our investigation. Figure 4A showed the predicted binding sites between miR‐629 and 3′UTR of FOXO3. Based on the predicted sites, the luciferase reporter vectors containing the wild‐type 3′UTR of FOXO3 or mutated 3′UTR of FOXO3 were constructed by the pGL3 plasmids. The dual‐luciferase reporter assay showed that overexpression of miR‐629 suppressed the luciferase activity of reporter vectors containing wild‐type 3′UTR of FOXO3, but not the ones containing the mutated 3′UTR of FOXO3 in HPASMCs (Figure 4B,C). On the other hand, miR‐629 inhibition increased the luciferase activity of the reporter vectors containing wild‐type 3′UTR of FOXO3, but not the ones containing the mutant one in hypoxia‐treated HPASMCs (Figure 4D,E). Consistently, miR‐629 mimics transfection also significantly suppressed the expression of FOXO3 mRNA and protein as revealed by qRT‐PCR and western blot assays (Figure 4F,G). The expression of FOXO3 mRNA and protein was suppressed in HPASMCs after exposing to hypoxia compared to cells under normoxia conditions (Figure 4H,I).

**Figure 5:**
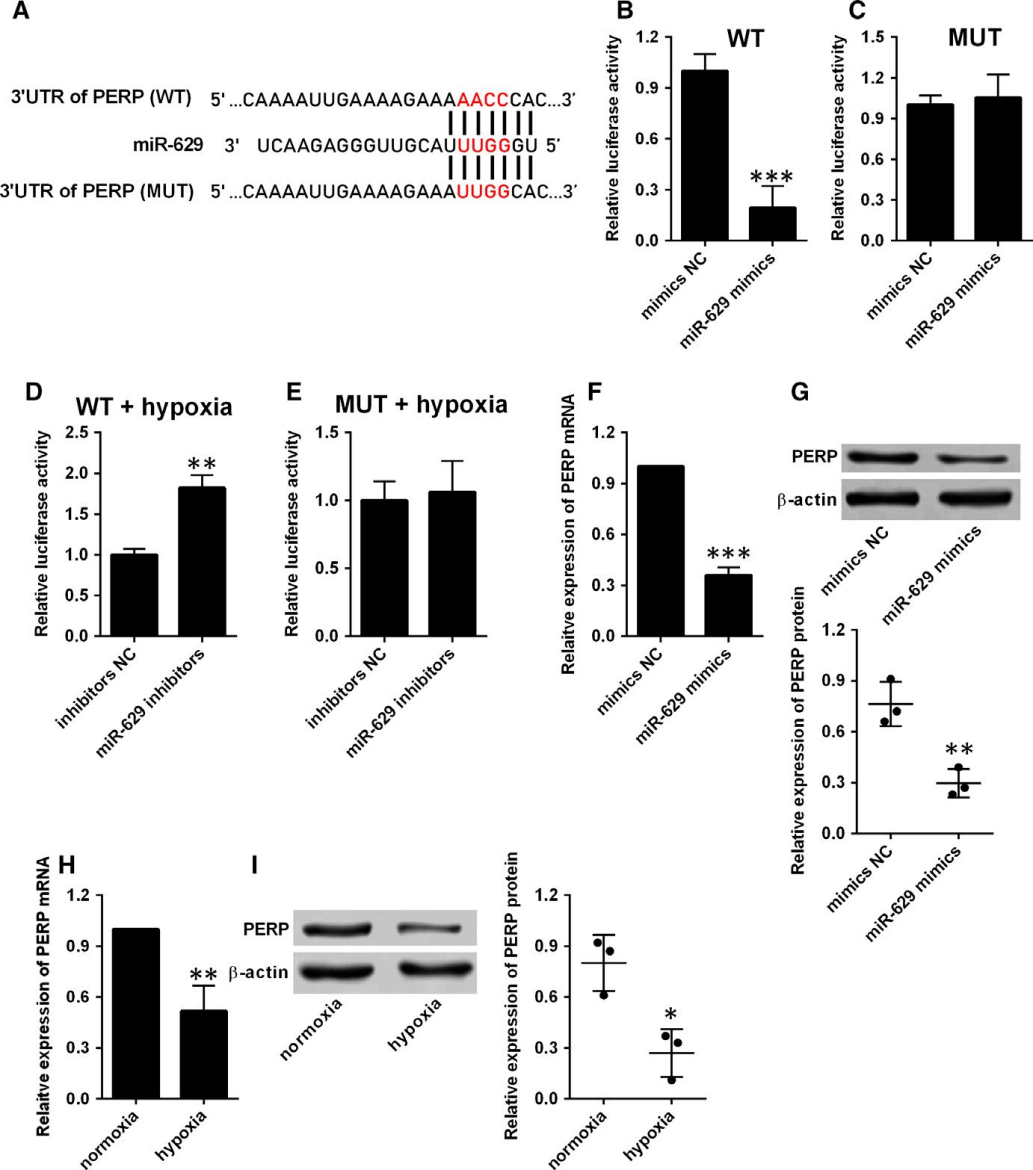
MiR‐629 directly targets PERP. A, The predicted binding sites between 3′UTR of PERP and miR‐629 as determined by the targetscan software. B, The relative luciferase activity of the reporter vector containing wide‐type (WT) 3′UTR of PERP was determined at 48 h after transfection with mimics NC or miR‐629 mimics. C, The relative luciferase activity of the reporter vector containing mutant (MUT) 3′UTR of PERP was determined at 48 h after transfection with mimics NC or miR‐629 mimics. (D and E) The relative luciferase activity of the reporter vector containing mutant 3′UTR of PERP was determined at 48 h after transfection with inhibitors NC or miR‐629 inhibitors in hypoxia‐treated HPASMCs. (F and G) The expression of PERP mRNA and protein in HPASMCs after being transfected with mimics NC or miR‐629 mimics was determined by qRT‐PCR and western blot respectively. (H and I) The expression of PERP mRNA and protein in HPASMCs after exposing to hypoxia or normoxia was determined by qRT‐PCR and western blot respectively. Data are expressed as mean ± standard deviation; n = 3. *P < 0.05, **P < 0.01 and ***P < 0.001 versus control groups

Similarly, based on the predicted sites shown in Figure 5A, the luciferase reporter vectors containing the wild‐type 3′UTR of PERP or mutated 3′UTR of PERP were constructed by the pGL3 plasmids. Overexpression of miR‐629 suppressed the luciferase activity of wild‐type reporter vectors but not the mutated ones (Figure 5B,C). On the other hand, miR‐629 inhibition increased the luciferase activity of the reporter vectors containing wild‐type 3′UTR of PERP, but not the ones containing the mutant one in hypoxia‐treated HPASMCs (Figure 5D,E). Overexpression of miR‐629 also induced a decrease in the mRNA and protein expression levels of PERP in HPASMCs (Figure 5F,G). Furthermore, hypoxia treatment also suppressed the mRNA and protein expressions of PERP in HPASMCs when compared to normoxia group (Figure 5H,I).

### 3.5 Overexpression of FOXO3 and PERP reversed the effects of miR‐629 overexpression on cell proliferation, migration and apoptosis of HPASMCs

**Figure 6:**
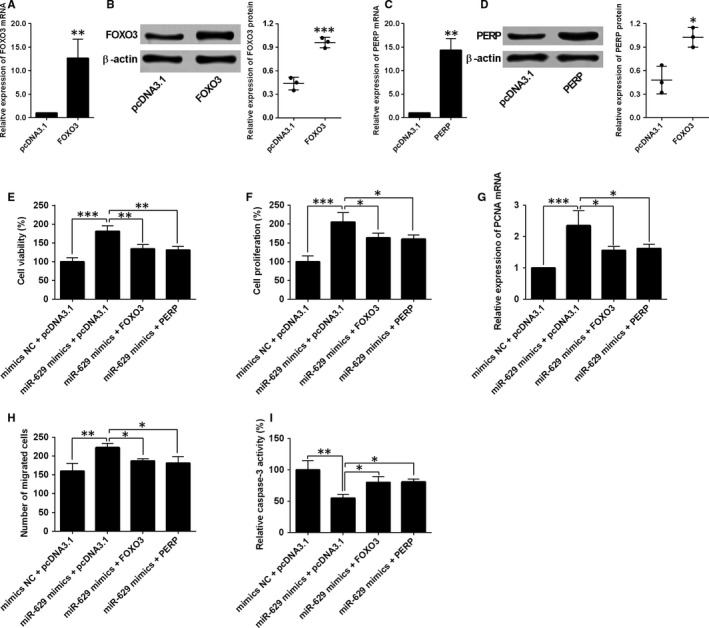
Overexpression of FOXO3 and PERP reversed the effects of miR‐629 overexpression on cell proliferation, migration and apoptosis of HPASMCs. (A and B) The expression of FOXO3 mRNA and protein in HPASMCs after being transfected with pcDNA3.1 or pcDNA3.1‐FOXO3 was determined by qRT‐PCR and western blot respectively. (C and D) The expression of PERP mRNA and protein in HPASMCs after being transfected with pcDNA3.1 or pcDNA3.1‐PERP was determined by qRT‐PCR and western blot respectively. (E) Cell viability and (F) cell proliferation of HPASMCs after being co‐transfected with miRNAs and plasmids were determined by CCK‐8 assay and BrdU assay respectively. G, The expression of PCNA mRNA in HPASMCs after being co‐transfected with miRNAs and plasmids was determined by qRT‐PCR. H, Cell migration of HPASMCs after being co‐transfected with miRNAs and plasmids was measured by transwell migration assay. I, Cell migration of HPASMCs after being co‐transfected with miRNAs and plasmids was measured by transwell migration assay. Data are expressed as mean ± standard deviation; n = 3. ^*^ *P* < 0.05, ^**^ *P* < 0.01 and ^***^ *P* < 0.001 versus control groups

To determine whether miR‐629 exerted its effects via FOXO3 and PERP in HPASMCs, we performed the rescue experiments by overexpressing FOXO3 or PERP in HPASMCs. The overexpression of FOXO3 or PERP was performed by transfecting HPASMCs with the respective plasmids that overexpress FOXO3 or PERP (Figure 6A‐D). The CCK‐8 assay and BrdU assay showed that enforced expression FOXO3 or PERP attenuated the enhanced effects of miR‐629 overexpression on the cell viability and proliferation of HPASMCs (Figure 6E,F). The up‐regulation of PCNA induced by miR‐629 overexpression was also attenuated by the enforced expression of FOXO3 or PERP (Figure 6G). In addition, enforced expression of FOXO3 or PERP also prevented the increase in the number of migrated HPASMCs induced by miR‐629 overexpression (Figure 6H). The Caspase‐3 activity assay showed that a decrease in the caspase‐3 activity caused by miR‐629 overexpression was partially reversed by enforced expression of FOXO3 or PERP in HPASMCs (Figure 6I).

### 3.6 Overexpression of FOXO3 and PERP reversed the hypoxia‐induced effects on cell proliferation, migration and apoptosis of HPASMCs

**Figure 7:**
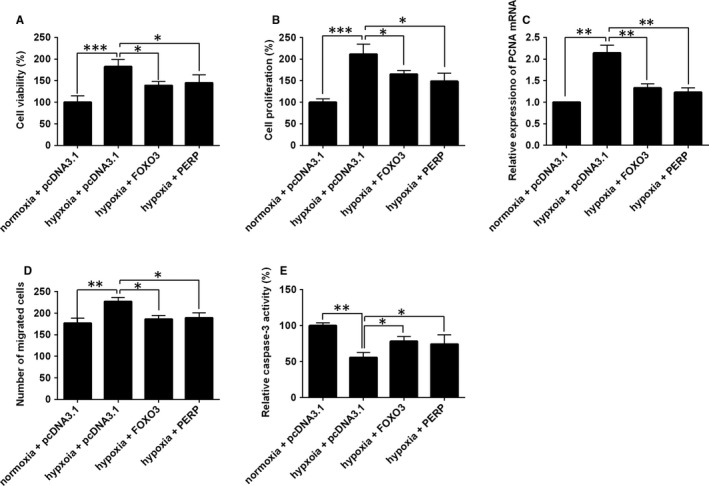
Overexpression of FOXO3 and PERP reversed the hypoxia‐induced effects on cell proliferation, migration and apoptosis of HPASMCs. HPASMCs under the normoxia condition served as negative controls. In other groups, HPASMCs were treated with hypoxia for 48 h, (A) cell viability, (B) cell proliferation, (C) expression of PCNA mRNA, (D) cell migration and (E) caspase‐3 activity were determined by respective in vitro functional assays at 24 h post‐transfection with pcDNA3.1, pcDNA3.1‐FOXO3 or pcDNA3.1‐PERP. Data are expressed as mean ± standard deviation; n = 3. *P < 0.05, **P < 0.01 and ***P < 0.001 versus control groups

The rescue experiments were further performed to address if FOXO3 or PERP mediated the hypoxia‐induced changes in the cellular functions of HPASMCs. As shown in Figure 7A‐C, enforced expression of FOXO3 or PERP partially reversed the hypoxia‐induced increase in cell viability, proliferation and expression level of PCNA mRNA in HPASMCs (Figure 7A‐C). Consistently, hypoxia treatment increased the migratory potential of HPASMCs, and this effect was reversed by the enforced expression of FOXO3 or PERP (Figure 7D). In addition, enforced expression of FOXO3 or PERP also attenuated the suppressive effects of hypoxia treatment on the caspase‐3 activity in HPASMCs (Figure 7E).

### 3.7 The expression of miR‐629, FOXO3 and PERP mRNA in clinical samples

**Figure 8:**
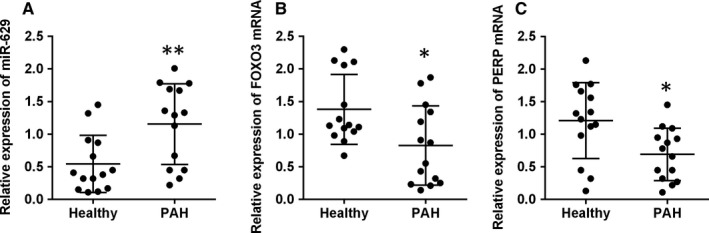
The expression of miR‐629, FOXO3 and PERP mRNA in clinical samples. A, The expression of miR‐629 in the plasma from healthy controls (n = 14) and PAH patients (n = 14) was determined by qRT‐PCR. B, The expression of FOXO3 mRNA in the plasma from healthy controls (n = 14) and PAH patients (n = 14) was determined by qRT‐PCR. C, The expression of PERP mRNA in the plasma from healthy controls (n = 14) and PAH patients (n = 14) was determined by qRT‐PCR. *P < 0.05 and **P < 0.01 versus control groups

The expression of miR‐629, FOXO3 and PERP mRNA in the plasma from healthy controls and PAH patients was determined by qRT‐PCR. As shown in Figure 8A, the expression of miR‐629 was up‐regulated in the plasma from PAH patients when compared to healthy controls. Consistently, the mRNA expression of FOXO3 and PERP was down‐regulated in the plasma from PAH patients when compared to healthy controls (Figure 8B,C).

## 4 DISCUSSION

This study is the first, to our knowledge, to identify up‐regulation of miR‐629 in the in vitro experimental models of HPASMCs exposing to hypoxia. Treatment with miR‐629 mimics promoted HPASMCs proliferation and migration, but inhibited cell apoptosis; while knockdown of miR‐629 suppressed the cell proliferation and migration but promoted cell apoptosis in hypoxia‐treated HPASMCs. The bioinformatics prediction revealed FOXO3 and PERP as downstream targets of miR‐629, and miR‐629 negatively regulated the expression of FOXO3 and PERP via targeting the 3′UTRs. Further rescue experiments showed that the hyper‐proliferation of HPASMCs induced by hypoxia may be related to miR‐629/FOXO3 and PERP signalling pathways. Furthermore, the expression of miR‐629 was up‐regulated, and the expression of FOXO3 and PERP mRNA was down‐regulated in the plasma from PAH patients when compared to healthy controls. The present results provide a mechanism by which miR‐629 expression may be inhibited for therapeutic benefit in PAH patients.

Despite great advances have been made in the treatment of PAH, the pharmacological treatment for PAH is still limited and far from satisfaction. Recently, miRNAs are found to involve in the vascular remodelling of PAH,^17^ suggesting targeting miRNAs may represent alternative approaches for the development of novel therapeutics for PAH. Studies demonstrated that hypoxia treatment induced miR‐1 up‐regulation which contributed to PASMC hypertrophy and a reduction in the Kv1.5 channels, suggesting the pathophysiological role in PAH.^18^ miR‐429 and miR‐42‐5p were found to inhibit HPASMC proliferation and Ca2+ influx by suppressing calcium sensing receptor expression.^19^ Yang et al, found that miR‐760 was down‐regulated in hypoxia‐induced HPASMCs and miR‐760 regulated hypoxia‐induced proliferation and apoptosis of HPASMCs via targeting toll‐like receptor 4.^20^ In our results, we showed that miR‐629 was up‐regulated in hypoxia‐induced HPASMCs. Previous studies also showed the up‐regulation of miR‐629 in several types of cancers, and overexpression of miR‐629 showed enhanced effects on the cancer cells including cervical cancer, colorectal cancer, breast cancer and ovarian cancer.^13^, ^14^, ^15^, ^16^ Consistently, our data showed that overexpression of miR‐629 promoted cell proliferation and inhibited cell apoptosis of HPASMCs, while knockdown of miR‐629 exerted the opposite effects in HPASMCs during hypoxia. Taken together, our results suggested the hyper‐proliferation of HPASMCs induced by hypoxia could be mediated via up‐regulation of miR‐629.

To further address the mechanistic role of miR‐629 in the regulation of HPASMCs proliferation, we identified two potential genes (FOXO3 and PERP) as the targets of miR‐629, and FOXO3 and PERP were negatively regulated by miR‐629 in HPASMCs. FOXO3 belongs to the superfamily of the mammalian forkhead box O transcriptional factors, which has been shown to play important roles in cancer, metabolism, ageing and vascular homoeostasis.^21^ Studies demonstrated that FOXO3 functionally interacted with the PI3K/AKT signalling pathway to regulate the proliferation of smooth muscle cells.^22^ In addition, activation of FOXO3 contribute to the protective effects of salidroside on hypoxia/reoxygenation‐induced human brain vascular smooth muscle cell injury.^23^ FOXO3 was down‐regulated in the fibroblasts isolated from PAH calves and patients with idiopathic PAH and functioned as an important mediator in the miR‐124‐mediated proliferation of pulmonary vascular fibroblasts,^24^ suggesting the potential role of FOXO3 in the pathophysiology of PAH. PERP was found to function as a p53 transcriptional target that is induced during apoptosis in response to DNA damage.^25^ PERP was found to suppress tumour cell proliferation and promote cell apoptosis in various types of cancers.^26^, ^27^, ^28^ Additionally, silencing of PERP was found to inhibit the cell apoptosis in hypoxia‐mediated injury in renal cells.^29^ As expected, our data showed that enforced expression of FOXO3 or PERP attenuated the miR‐629 overexpression or hypoxia‐induced enhanced effects on HPASMC proliferation and suppressive effects on HPASMC apoptosis, suggesting that hypoxia‐induced changes could be possibly mediated via miR‐629/FOXO3 and PERP signalling pathways.

There are still several limitations in the present study. First of all, experiments of this study were only focused on the in vitro assays to reveal the mechanistic actions of miR‐629 in the hypoxia‐treated HPASMCs, which is a limitation of the present study. As such, a PAH animal model should be employed to further look into the in vivo actions of miR‐629 in pathophysiology of PAH. Though the expression of miR‐629, FOXO3 and PERP determined the clinical samples, the sample size was relatively small and the samples were collected in single centre. In this regard, more samples and multicentre studies should be employed to consolidate our current findings. Moreover, as miRNAs could target many genes, and this study only investigated FOXO3 and PERP, further studies may explore other potential targets to fully elucidate the regulatory mechanisms of miR‐629 in PAH.

In conclusion, our results showed that miR‐629 was up‐regulated in HPASMCs during hypoxia, and knockdown of miR‐629 attenuated hypoxia‐induced cell proliferation and migration as well as the suppressive effects of hypoxia on cell apoptosis in HPASMCs. Further mechanistic studies revealed that the hypoxia‐induced changes in PASMC proliferation, migration and apoptosis may involve the miR‐629/FOXO3 and PERP signalling pathways. The present study provided evidence regarding the novel role of miR‐629 in regulating cell proliferation, migration and apoptosis of HPASMCs during hypoxia.

## CONFLICT OF INTEREST

The authors declare that there are no conflict of interest.
